# Neuromotor control of the triceps surae during running in people with and without Achilles tendinopathy and the immediate effect of foot orthoses

**DOI:** 10.1186/1757-1146-4-S1-O52

**Published:** 2011-05-20

**Authors:** Narelle Wyndow, Sallie Cowan, T Wrigley, Kay Crossley

**Affiliations:** 1 Center for Health Exercise and Sports Medicine, Melbourne Physiotherapy School, The University of Melbourne, Melbourne, Victoria, 3010, Australia; 2 Melbourne Physiotherapy School, Melbourne, Australia, 3010, Australia; 3 Department of Engineering, Melbourne School of Engineering, Melbourne, Victoria, 3010, Australia

## Background

Achilles tendinopathy (AT) is a common injury among physically active populations. It has been proposed that changes in neuromotor control of the triceps surae may increase differential intratendinous forces and thus be associated with the pain and pathology seen in this condition. However, it is not known if neuromotor differences actually exist between those with and without this condition. Thus the primary purpose of this research was to investigate whether neuromotor control of the triceps surae in distance runners with AT is altered compared to controls (Study 1). The secondary purpose of this research was to investigate the immediate effects of foot orthoses on triceps surae neuromotor control in subjects with AT (Study 2).

## Methods

Surface electromyographic measures were taken from the Soleus (Sol), Lateral Gastrocnemius (LG) and Medial Gastrocnemius (MG) of 34 male subjects (15 with AT, 19 controls) while subjects ran over ground at 4m/sec in a running sandal. Force plate data was acquired to determine heel strike and toe off events. For Study 1, comparisons were made between the relative timing of each of the three muscles for EMG onset and offset, i.e. Sol-LG. Sol-MG and LG-MG. For study 2, the same measures were taken while subjects with AT ran in a prefabricated orthoses.

## Results

For study 1, there was a significant difference for Sol and LG offset times in the AT group, compared to the control group(p = 0.02). There were no significant differences for EMG onset times between groups. For study 2, no significant differences were found in the AT group between the footwear only condition and the footwear plus orthoses condition (p > 0.05).

## Conclusions

Subjects with AT display altered neuromotor control of the triceps surae compared to controls. Sol offset times were significantly earlier than LG offset times. It is not known whether this is as a result of the pathology or is an aetiological factor in the genesis of AT. Foot orthoses had no immediate effect on the relative timing of the triceps surae. Further research is required to understand the genesis of the neuromotor differences and to determine whether there are any long-term responses to foot orthoses.

